# Physical Activity, Trust, and Research Participation Among Men From Minority Ethnic Backgrounds Living With Prostate Cancer: A Qualitative Study

**DOI:** 10.1002/pon.70408

**Published:** 2026-02-24

**Authors:** Jack Carr, Mark A. Faghy, David Broom, Clare Roscoe, Kevin Williams, Ruth E. M. Ashton

**Affiliations:** ^1^ Research Centre for Physical Activity Sport and Exercise Sciences (PASES) Coventry University Coventry UK; ^2^ Biomedical and Clinical Exercise Science Research Theme University of Derby Derby UK; ^3^ School of Sport Exercise and Health Sciences Loughborough University Loughborough UK; ^4^ University Hospitals Coventry and Warwickshire (UHCW) Coventry UK

## Abstract

**Objective:**

Men from minority ethnic backgrounds experience a disproportionate burden of prostate cancer yet remain underrepresented in physical activity‐related and psycho‐oncology research. This study aimed to explore (1) how men from diverse ethnic backgrounds experience and interpret physical activity (PA) following prostate cancer, and (2) how psychological, cultural, and structural factors influence their engagement with PA and research.

**Methods:**

Semi‐structured interviews were conducted with ten men from African, Caribbean, Asian, and Middle Eastern backgrounds living with prostate cancer. Sampling continued until thematic saturation was achieved, consistent with qualitative methodological guidance. Data was analysed using Braun and Clarke's reflexive thematic analysis. A patient‐informed topic guide and culturally reflexive approach were used to ensure contextual sensitivity and psychological safety.

**Results:**

Six interconnected themes were identified: (1) PA as Mental Renewal, Identity, and Connection; (2) Cancer‐Related Disruption and Fragmented PA Support; (3) Barriers to Participation in PA and Research; (4) Trust, Representation, and Inclusive Research Practices; (5) Cultural Stigma, Silence, and Shifting Perspectives; and (6) Altruism, Legacy, and Motivation to Engage. PA was described as psychologically meaningful, supporting coping, identity, and continuity, but was frequently disrupted by inconsistent guidance and structural barriers. Trust, representation, and relational communication were central to research engagement. Findings informed the development of a Culturally Sensitive Recruitment Framework.

**Conclusions:**

PA engagement and research participation among minority ethnic men with prostate cancer are shaped by intersecting psychological, cultural, and structural factors. Culturally sensitive, relationship‐centred approaches may strengthen integrated psycho‐oncology care and promote more equitable research participation.

## 1 Introduction

The Urology Foundation (TUF) [1] reports approximately 78,000 new urological cancers in the UK each year, with approximately 63,000 of those being prostate cancers [2]. Globally, 40% of all cancer diagnoses in men relate to urological cancer, and 29% of those diagnoses are linked to prostate cancer (PCa) [3]. According to the Global Cancer Observatory, prostate cancer alone accounted for 7.3% of all cancer cases in 2020, with varying incidence rates across regions and ethnic groups [4].

Global cancer incidence is highest in Black men, largely driven by PCa. PCa incidence is 70% higher in Black than White men [3]. In addition, mortality rates are twice as high in Black men diagnosed with prostate cancer compared to White men [5]. This disparity between ethnic groups is also evident in the UK, where Black men are at twice the risk of both diagnosis and mortality from PCa compared to their White counterparts [6, 7]. Delon et al. [7] also found that rates of bladder, kidney, testicular and penile cancer were lower or similar in non‐White minority ethnic groups compared with the White ethnic group.

According to a recent systematic review in England and Wales, the demographic composition predominantly comprises 86.9% White individuals, 7.14% Asian individuals, and 2.36% Black individuals [8]. Within the study by Delon et al. [7], 9599 White individuals were diagnosed with kidney cancer, compared with a smaller cohort of 1215 individuals from other ethnic minority groups. This imbalance highlights important limitations related to population representation and the interpretation of ethnic differences in outcomes.

According to Siegal et al. [3] survival rates for kidney cancer between White and Black individuals are similar. However, kidney cancer survival is lower in Black people for every histologic subtype of the disease (26.3% vs. 18.4%). Overall survival appears similar because Black people have a higher proportion of papillary and chromophobe renal cell carcinoma (RCC) (12.5%) compared to White people (4.5%), subtypes associated with a better prognosis than other types of RCC [9]. This highlights data previously collected can be potentially misleading, demonstrating the need for targeted research to understand and address health inequalities.

Against this backdrop of marked ethnic disparities in urological cancer burden and outcomes, PA represents a promising, low‐cost intervention with potential to address both clinical and psychosocial inequalities.

The NICE currently recommends PA but has no published guidelines specifically addressing PA during or after cancer treatment [10]. This is despite several studies demonstrating that PA is safe and can improve cancer related reported outcomes following treatment [11, 12]. PA during and after prostate and bladder cancer treatment has been shown to be safe and alleviate treatment associated side‐effects such as fatigue, nausea, vomiting, erectile dysfunction and loss of libido, while improving health measures such as blood pressure, aerobic capacity, and body composition [13, 14, 15]. However, the applicability of these findings to diverse ethnic groups remains an underexplored and underrepresented area. Making focused research to elucidate the perspectives of minority ethnic populations on their experience of undertaking PA as an integral component of urological cancer care.

Despite clear ethnic differences in cancer incidence and outcomes, there remains a critical lack of dedicated studies that explore the unique experiences, challenges, and perspectives of individuals from minority backgrounds affected by urological cancers. Existing evidence is overwhelmingly derived from White populations, limiting insight into how cultural beliefs, identity, stigma, and structural barriers shape engagement with exercise and research participation among ethnically diverse men. According to McAllister [16] demonstrated that while searches for PCa yielded large quantities of paper responses, when keywords such as ‘ethnic minority’ or ‘African‐Caribbean’ were added, the numbers of papers returned dramatically reduced. Specifically, results decreased from 180,000 results to 140 results, underlining a universal lack of investigation into the association between ethnicity and PCa. Further studies highlight that approximately 96% of men participating in PCa research are White and that 80% of PCa trials registered on clinicaltrials.gov, funded by pharmaceutical or biotechnology companies, included a higher proportion of White men than the publicly funded trials [17, 18]. A similar finding has also been highlighted in studies with male bladder and kidney cancer patients, where Black and Hispanic patients have been underrepresented compared to White counterparts [19].

Such limited heterogeneity has substantial implications for clinical practice and equity. When the evidence underpinning PCa care is derived almost entirely from White cohorts, it restricts our understanding of how biological, cultural, structural, and behavioural factors shape outcomes for ethnically diverse men. This not only undermines the external validity of health research but perpetuates inequities by producing interventions and recommendations that may not translate effectively across communities with differing lived experiences, health beliefs, and access to care. Addressing this gap is essential not only for improving the inclusivity and external validity of psycho‐oncology research, but also for informing the development of culturally responsive, integrated care pathways that promote equity in both clinical outcomes and research participation.

The primary aim of this research was to investigate enabling factors influencing participation in PA within the urological cancer population from diverse ethnic backgrounds. By investigating themes such as current PA behaviour and attitudes toward PA will give us important insights to optimise interventions.

The inclusion of minority ethnic groups in health research is essential for ensuring the generalisability of research findings and the development of tailored interventions that consider diverse experiences. Yet, disparities persist in the representation of ethnic minorities in health research. To address these gaps the secondary aim is to investigate the participants experiences with research participation, to generate context‐specific insights that can inform more targeted and inclusive approaches to cancer care and research recruitment.

## 2 Methods

NHS ethical approval (IRAS: 345005) was granted by the London‐Camberwell St. Giles REC. Institutional ethical approval by Coventry University's Research Ethics Committee [P178645]. The study was also conducted in accordance with the Helsinki Declaration [20].

### 2.1 Patient and Public Involvement and Engagement

Patient and public involvement and engagement (PPIE) was incorporated in an advisory and consultative capacity throughout the design and delivery of this study, rather than through formal co‐production.

Prior to and during recruitment, the research team engaged with members of the public and individuals with lived experience of cancer through community outreach events, as well as through local cancer support groups and charities. Examples of outreach events include The Coventry Caribbean Association; The Arawak Community Trust (ACT); and The Coventry and Warwickshire Cancer Bus Tour organised by UHCW and delivered in partnership with Macmillan Cancer Support. Conversations with these groups provided valuable insight into culturally appropriate ways of approaching potential participants, preferred communication methods, style and language, and common barriers to research participation within minority ethnic communities.

This feedback directly informed refinements to the recruitment strategy and interview design, including the emphasis on face‐to‐face engagement and conversational approaches. Ongoing engagement with community organisations (including The Coventry Caribbean Association, ACT, and The Black Health Initiative UK) also helped to refine recruitment messaging and build trust between the research team and potential participants, ensuring that the study remained grounded in the lived realities and preferences of those it aimed to represent. Findings from this project will be shared with these community partners to support continued dialogue and co‐design of future research.

### 2.2 Study Design

Semi‐structured interviews were used to explore perceptions of PA, research participation, and clinical engagement among minority ethnic men living with prostate cancer. Interviews were conducted by one researcher with experience and qualification in qualitative research and conducting semi‐structured interviews (JC).

While the original protocol proposed the use of focus groups, recruitment challenges and participant preference led to a pragmatic adaptation to change to one‐to‐one interviews. This format was deemed more appropriate for discussing sensitive topics such as cancer, masculinity, and cultural stigma, while also accommodating participants who preferred a more private setting to avoid interacting with others.

Recruitment was facilitated through word‐of‐mouth at community cancer awareness events, links established through PPIE, and through clinical contacts on site and in partnership with University Hospitals Coventry and Warwickshire (UHCW), Coventry, UK. Eligible participants received a Participant Information Sheet (PIS) detailing the study aims, procedures, risks, and benefits, and provided written informed consent prior to participation. All participants were reminded of their right to withdraw at any time without consequence. Ten participants with ethnic backgrounds including Iran, India and Afro‐Caribbean, aged between of 53 and 70, were recruited between April and October 2025. Participants that were recruited were currently living with or had previously had a prostate cancer diagnosis at some point in their lives. Recruitment challenges, including the frequency of participant recruitment which affected sample size, also reinforced the rationale for an adaptation from focus groups to one‐to‐one interviews.

### 2.3 Eligibility Criteria

Participants were considered eligible for inclusion in this study if they met the following inclusion criteria: (1) Aged 18 years and over with a confirmed diagnosis of prostate cancer by a healthcare clinician; (2) Participants representing minority ethnic backgrounds in the context of the UK, including but not limited to: African/African‐Caribbean, Asian, Hispanic/Latino, and Middle Eastern; (3) Participants who expressed a willingness to participate in a semi‐structured interview and discuss their experiences of physical activity, cancer care, and research participation; (4) Ability to communicate in English. Participants who do not speak English are permitted to have a family member present during interviews and consultations to translate research documents, and act as translator during interviews.

### 2.4 Demographic and PA

Prior to interview, participants completed a brief demographic and health questionnaire to glean their age, ethnicity, cancer type, treatment details, comorbidities, smoking status, and current PA behaviour. The International Physical Activity Questionnaire (IPAQ) Short Form was used to assess PA behaviour in the previous week and provide contextual insight into participants' PA engagement. The IPAQ Short Form has demonstrated acceptable reliability (Spearman's *ρ* = 0.76) and validity against accelerometer measures [21].

While device‐based measures such as accelerometers can offer more objective estimates of PA, their use was not feasible in this study due to practical constraints related to participant accessibility, digital literacy, and the community‐based recruitment approach. The IPAQ Short Form was therefore selected as a validated, low‐burden tool suitable with qualitative study designs where the primary focus was on contextual and experiential insights rather than precise quantification of PA levels.

### 2.5 Interview Procedure

Semi‐structured interviews were guided by a topic guide to ensure consistency across interviews. All participants were asked broadly the same questions covering key domains. However, the order and phrasing of questions were flexibly adapted during interviews in response to participants' responses, allowing the interviewer to probe emergent topics and clarify meaning where appropriate. Interviews lasted between 45 and 60 min, and were conducted either in person, via Microsoft Teams, or by telephone, according to participant preference. The topic guide was developed and reviewed by the author team. The topic guide was refined iteratively during the early stages of data collection. Feedback from the first participant led to the inclusion of a question inviting individuals to describe their cultural background in their own words. This allowed participants to express their cultural and/or ethnic identity in their own terms, offering space for nuance and self‐definition rather than categorisation within pre‐set ethnic labels. This adaptive approach enabled the guide to remain responsive to participant feedback and to capture more authentic, contextually grounded accounts. The final topic guide was then used to explore key areas including current PA habits and attitudes; the perceived role of PA within cancer care; participant experiences and perceptions of clinical trial participation; barriers and facilitators to engagement in research and PA programmes, and cultural attitudes influencing openness, stigma, and trust in healthcare (See Table S1).

### 2.6 Data Analysis

All interviews were conducted, audio‐recorded (using either the record function in Microsoft Teams or the Voice Memos iOS app), transcribed using intelligent verbatim by one researcher (J.C.), and a percentage (30%) of audio recordings were cross‐checked against the transcriptions for accuracy by another (R.A.) [22, 23].

Interview questions were designed to foster open, participant‐led discussion. For one participant with epilepsy who required a family member to act as an interpreter, the interview was adapted to ensure comfort and accessibility while maintaining ethical safeguards.

Data collection continued until thematic saturation was achieved according to guidelines by Guest et al. [24], who define saturation as the point at which no new themes, codes, or insights emerge from successive interviews, and further data collection is unlikely to yield additional conceptual information.

The general inductive thematic analysis approach was used to identify themes in the text data relating to the study objectives, following Braun and Clarke's [25] thematic analysis framework. Once familiarised with the data, initial codes were generated by one reviewer (J.C.). After this, themes identified were then reviewed collaboratively with the wider research team (R.A., M.F., D.B.) to quality check, enhance reliability, and credibility.

Following good practice guidelines outlined by Smith and McGannon [26], steps were taken to minimise power imbalances between the researcher and participants. The interviewer adopted a conversational and empathetic stance, encouraging participants to lead the discussion and share their experiences in their own words. The interviewer also drew on shared social and cultural understanding where appropriate to foster rapport and trust. This approach aimed to position participants as experts in their own lived experiences.

Member checking was not undertaken, as the study sought to capture spontaneous reflections and emotional responses, which may have been altered retrospectively. Additionally, given the sensitivity of the topic and the potential for participants to re‐engage with distressing experiences, follow‐up contact was deemed ethically inappropriate and inconsistent with the study's low‐burden design. Instead, analytic rigour was ensured through iterative coding discussions within the research team, reflexive journaling, and transparent documentation of coding decisions throughout the analysis process.

### 2.7 Positionality and Reflexivity Statement

The lead researcher (JC) is a PhD student researching exercise oncology with a background in clinical exercise physiology. His prior experience in exercise and cancer research may have shaped his interest in the topic, and this familiarity may have influenced data collection and interpretation, including a potential inclination to view exercise positively or to assume shared understandings regarding the benefits of PA among participants.

At the same time, this background facilitated rapport with participants, particularly when discussing exercise behaviours, treatment‐related side effects, and interactions with healthcare services. Participants often engaged openly with discussions of PA, training adaptations, and recovery, which may have been supported by the researcher's ability to communicate using accessible, experience‐informed language.

To mitigate potential bias and avoid privileging biomedical or exercise‐centric interpretations, the researcher engaged in ongoing reflexive practices, including reflective journaling and supervisory debriefing were used throughout the research process to maintain awareness of potential biases. The researcher approached interviews with openness, aiming to ensure participants' perspectives were foregrounded over researcher assumptions.

## 3 Results

### 3.1 Participant Characteristics

Ten participants took part in the study, all of whom had been diagnosed with prostate cancer at some point in their lives. Each participant was invited to complete an online demographic questionnaire via Qualtrics prior to their interview to record information such as age, ethnicity, and occupational status.

Eight participants completed the questionnaire in full prior to the interview. Despite non‐completion from two participants, deeper information was obtained for all ten participants through questioning during the interview. The sample comprised one participant of Iranian backgroound, two of Indian background, three of African background (two Nigerian Yoruba; one Cameroonian), and four of Caribbean background (all Jamaican). Participant characteristics obtained from the Qualtrics responses are summarised in Table 1.

**TABLE 1:** Participant characteristics from qualtrics questionnaire responses (*n* = 8).

Participant characteristic (*n* = 8)	
Age (years), mean ± SD (range)	62.9 ± 6.0 (53, 70)
Ethnicity:	
Black/Black British/Black Welsh (Caribbean or African)	6
Asian/Asian British/Asian Welsh	2
Smoking history:	
Ex‐Smoker	1
Never	7
Occupational status:	
Employed	2
Unemployed	1
Full‐time student	1
Retired	4
Height (cm) (min, max)	177.6 (165, 198)
Weight (kg) (min, max)	87 (58, 110)
Comorbidities:	
High blood pressure	1
High cholesterol	1
Epilepsy	1
None	5
Cancer contained to prostate:	
Yes	4
No	3
Don't know	1
Treatment type:	
Prostatectomy/surgery	4
Radiotherapy and/or Hormone Ablation	3
Six‐monthly check‐up only	1

## 4 Results and Discussion

To avoid repetition, the findings and quotes from this novel study will be presented alongside the discussion and interpretation. Initially agreed themes will be presented.

### 4.1 Themes

**FIGURE 1:**
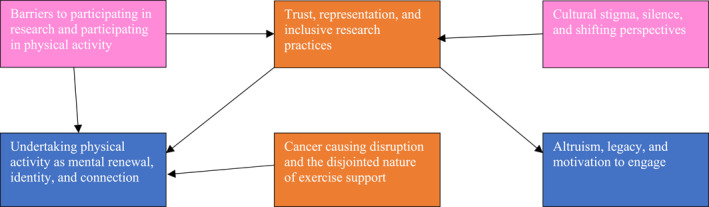
Thematic diagram showing each theme identified in the thematic analysis. Themes are colour‐coded to show what level each theme is at and arrows coming from on theme to another show directional influences between themes. Code for theme levels is Pink = structural and cultural level; Orange = interpersonal level; and Blue = individual level.

Thematic analysis generated six interrelated themes that captured how men from minority ethnic backgrounds understood, experience, and engage with PA and research participation following a prostate cancer diagnosis (See Figure 1).

Collectively, these themes illustrate that PA held physical, psychological, and social meaning for participants. PA offered mental renewal, a sense of identity, and connection, yet opportunities to sustain this were often interrupted by sporadic support systems and unequal access to resources. Structural and logistical barriers intertwined with deeper cultural factors such as stigma, mistrust, and the lack of inclusive research spaces, ultimately compounding the challenges men faced in accessing support and engaging with research opportunities.

Across interviews, the importance of representation, face‐to‐face contact, and culturally tailored approaches emerged as key facilitators of engagement. Participants frequently expressed altruistic motivations to contribute to research and help future generations, reflecting a strong sense of community responsibility and desire for lasting impact. Together, the findings highlight how cultural identity, lived experience, and trust intersect to shape both PA participation and willingness to engage in research.

#### 4.1.1 Theme 1—Undertaking Physical Activity as Mental Renewal, Identity, and Connection

PA emerged as a personal and multi‐faceted experience that extended beyond being physically active to encompass strength, renewal, and identity. For some, particularly those of Caribbean heritage, PA was not a task but a way of being. PA reaffirmed self‐worth and continuity in the face of illness. As one participant reflected:

It’s just fun. Enjoyment. Love. That’s me.

(P005)

Another described how being active helped him *get in a good mental space* and *settles [his] mental anguish* after treatment *(P008)*.

Several men highlighted the psychological renewal and agency that came with movement, viewing PA as an affirmation of capability and normality amid physical uncertainty. For others, especially those navigating recovery, informal activity was redefined as a meaningful part of staying active and maintaining independence:

Before I was getting it wrong like this is going to gym and things like that… now I try as much as possible to do some things at home like washing, walking up to the toilet, cutting the grass… it’s part of exercise.

(P006)

Similarly, integrating PA into daily routines offered a sense of continuity and control, as other participants explained:

‘When I walk it helps me mentally and physically. It’s my one hour me time’ (P010), ‘I live close to a Country Park, so I enjoy the walk through the park and especially after work’.

(P003)

Overall, PA represented more than physical rehabilitation, it was central to identity, self‐expression, and resilience. Across accounts, participants framed PA as a bridge between previous and present selves, and as a means of reclaiming normalcy following cancer treatment. This underscores how, within these communities, PA functions not only as a health behaviour but as an act of emotional and psychological renewal.

Participants' described PA as part of identity and psychological renewal echoes a growing body of research highlighting the holistic benefits of PA for men with prostate cancer. Previous studies describe PA as a pathway to reclaiming control, purpose, and masculinity during survivorship [27, 28, 29]. Our findings are similar but extend these by showing that such meanings could further be shaped by cultural background, particularly among Caribbean participants who viewed PA as intrinsic to self and community life, rather than as a prescribed intervention. This emphasis on PA as restorative identity work rather than rehabilitation resonates with broader work on the psychosocial role of PA in chronic illness [30, 31] yet has rarely been explored through a cross‐cultural lens.

While PA provided participants with a vital sense of identity, continuity, and mental renewal, the onset of cancer and the accompanying treatment disrupted these routines, revealing gaps and inconsistencies in the support available for maintaining PA during the cancer care pathway.

#### 4.1.2 Theme 2—Cancer Causing Disruption and the Disjointed Nature of Exercise Support

Participants described how their diagnosis and treatment of cancer disrupted their exercise routines, both physically and psychologically. Fatigue, surgical recovery, and treatment side effects often limited the ability to maintain previous PA levels:

Until I did my research, because they don't tell you these things, they tell you about the (main) side effects. They don't tell you (about less common side effects) unless you call and say you have some side effects.

(P008)

For some, post‐treatment periods involved a temporary cessation of PA, despite understanding its importance:

When I did get diagnosed, after the surgery I stop [exercising].

(P004)

Guidance from clinicians was inconsistent and often narrowly focused. Most participants reported receiving minimal instruction, primarily around Kegel exercises:

They only asked me to do Kegels… I managed to do it on my own by reading on the internet.

(P006)

Others described clear and helpful instructions, albeit instructions mainly relating to further pelvic floor exercises:

It was very good information… every time they talk about exercise, pelvic floors preparation, they give me leaflets… No, it was brilliant.

(P009)

Participants with prior PA experience tended to continue independently, while others relied on personal research to navigate PA:

I was told and I was googling that I need to do pelvic floor exercises for the operation… So, yeah, if there’s anything extra you’d advise, I will definitely follow it up.

(P010)

Despite these challenges, participants expressed a strong interest in ongoing support for maintaining activity during treatment:

Yes, he would be [keen to engage with exercise during treatment]. That was something that was missing [from consultations] right?.

(P001)

The combination of treatment‐related disruption, inconsistent guidance, and the reliance on self‐directed PA highlights a fragmented support system, leaving participants to bridge the gap between clinical advice and their own recovery needs.

The fragmented and inconsistent nature of PA advice described by participants reflects well‐documented gaps in exercise‐oncology integration [32, 33, 34]. Previous studies have noted that prostate cancer patients often receive generic guidance, with pelvic floor exercises being the only routinely recommended activity. Our data reaffirms this limitation and suggests that for men from minority ethnic backgrounds, the consequences of this disjointed support are intensified by communication barriers and lower access to culturally relevant information. In contrast to previous work that focuses on behavioural motivation, these findings foreground structural and systemic barriers, supporting calls for PA referral pathways that are standardised, culturally sensitive, and embedded throughout the cancer care continuum [35, 36].

Alongside fragmented exercise support, a parallel challenge emerged in participants' experiences of barriers to participating in research and maintaining PA, showing that navigating both clinical guidance and study involvement can be complex and demanding.

#### 4.1.3 Theme 3—Barriers to Participating in Research and Undertaking Physical Activity

Participants described a range of personal, practical, cultural, and structural barriers that limited engagement in both PA and research. Travel and convenience were frequently cited, with one participant stating, *Depends on what it is about, the time, the date and how it helps me (P009)*, while another noted, *The only thing [barrier] I can think of might be time… especially trials that aren't paid for and you've got bills (P003).* Local accessibility was also important:

Yeah again, my comfort zone, if it is locally, then I will do it… I think anything you like; I will go for it.

(P010)

Generational differences in PA participation were highlighted, with some participants observing that younger men, particularly in African communities, were more health‐conscious due to social influences:

For people my age, for older people my age, I think it's not very, very common. We live kind of sedimentary type of lifestyle and then we're kind of used to sitting down a lot… The younger generation like my son, my son goes to the gym regularly and he wants to show‐off his muscles….

(P002)

Others noted older generations were more physically active, reflecting cultural or familial habits:

‘In some way I can say older generation was more active than the younger’ (P009), ‘I think there is now and there is now, because I found that when I was younger there was a lot of opportunity to sort of do sport. There's probably more now, the younger ones who do not get involved in sport as much as we did when we were young’.

(P005)

Participants also described personal challenges, such as managing health conditions or sustaining PA routines:

‘Because of his epilepsy, he has a slight limp, so he doesn’t walk for long without a break, so there’s little cardio in his routine’ (P001), ‘I’m not very good at [exercise training] plans… I could do it for a while and then get switched off and do something else’.

(P002)

Barriers to research participation were similarly multifaceted. Scepticism or distrust about research motives was raised:

Don’t want to be a guinea pig… with the drug trial, what scares me is, I don’t like anything going in my body.

(P004)

Participants emphasised the need for clarity and relevance in research studies, as illustrated by P006, *It depends on the type of research… if it was something that will make me take the creams, I will not do it (P006)*. Technological or language barriers could further restrict engagement, as shown in this quote:

Emails or WhatsApp, I don't do them. I don't, I don't know that.

(P007)

Overall, participant accounts illustrate that barriers to PA and participating in research are intertwined with practical, social, and cultural factors. The data suggests that addressing engagement requires consideration of accessibility, culturally tailored information, and structural inequities, rather than focusing solely on individual motivation.

Consistent with the wider literature, participants identified cost, convenience, and accessibility as key barriers to PA [37, 38]. However, the present study adds further depth by showing how these intersect with cultural expectations, generational norms, and digital exclusion. Prior research suggests that minority ethnic patients are more likely to disengage when interventions are logistically demanding or presented through unfamiliar channels [39]. Our data illustrates how such barriers compound perceptions of research as inaccessible or irrelevant, highlighting the need for local, community‐based recruitment and delivery. These findings align with calls to ‘bring research to the people’ through partnerships with trusted community spaces such as faith centres, barbershops, and cultural organisations [40, 41].

The barriers participants described were not only logistical but also reflective of wider issues of trust, representation, and inclusivity within research and healthcare systems. This led to discussions around the importance of representation and culturally sensitive research practices.

#### 4.1.4 Theme 4—Trust, Representation, and Inclusive Research Practices

Participants demonstrated openness and enthusiasm toward research participation when trust, transparency, and personal relevance were established. Many described a willingness to engage if the purpose and benefits were clearly communicated. For example, P008 shared, *If there's a chance of it spreading and this can help eliminate it, I'd rather go for that… If you're just going to be something that's going to help for the future and I can be a part of it, why not?*. Similarly, P004 expressed, *It's nice to know there's possibilities… if I can help in any way.* Altruism was a recurring motivator across participants, with P007 simply stating their potential motivators for research participation would be *to help others.*

However, participants stressed that trust was not automatic, it depends on visibility, representation, and relational connection. P008 described feeling reassured when treated by a Black nurse who *“was relatable… she was able to put me at ease… consult me in terms of what would be best knowing what she knows about me as a Black man.”* This underscores how cultural understanding and representation can directly influence comfort and confidence in healthcare and research settings.

Many participants emphasised that recruitment methods mattered greatly for building this trust. P003 explained, *Face‐to‐face is better because interaction and questions can be asked and then some people are not technologically savvy, especially from ethnic minorities.* Similarly, P002 and P001 highlighted that community‐based approaches and trusted advocates were essential, with P002 noting that *there was actually a swimming event in the mosque that was organised in the mosque* and P001 suggesting that *leaders in the temples or the mosques, who have standing in the community can really help to reach the older generation.*

In contrast, written materials and digital methods were seen as ineffective or inaccessible for some. P003 mentioned that engagement through *committee events, maybe minority community events… or going to the GPs’*was a more effective route for inclusion. This was echoed by P010, who highlighted how representation affected engagement:

Whenever we start anything here [P010s place of work], when I tell my patient, like we are starting this new thing, they don’t take me seriously… But then I had my manager, she was White… engagement was better.

Some participants also reflected on how cultural ‘othering’ can discourage openness. P009 noted that ‘maybe based on ethnicity, it is much more I can say shameless or fear… you want to be in some place that people think of you differently,’ describing how stigma and fear of judgement within and outside their community could act as barriers.

Overall, participants viewed inclusive and relational research practices, such as diverse staffing and culturally relevant community outreach, as essential to fostering trust and participation. As P005 summarised, ‘Yeah definitely… if a clinical trial was specific to my ethnicity.’ These accounts show that inclusion in research is not merely about access but about belonging. Participants need to feel seen, understood, and represented.

The mistrust and scepticism toward research observed here mirrors historical and contemporary literature linking low participation to systemic inequities and underrepresentation [42, 43]. Our participants' emphasis on relational trust supports Ford et al. [40] argument that successful recruitment hinges on cultural congruence and visible diversity within research teams. Prior studies on racial concordance in healthcare [44] have shown that shared identity between patients and clinicians fosters confidence and communication; our findings extend this to research participation itself. Participants' rejection of impersonal digital outreach reflects longstanding critiques that institutional recruitment methods often fail to resonate with minority communities [45, 46].

While trust and representation emerged as key to fostering inclusion in research, participants also described how cultural stigma and silence surrounding illness could influence openness and engagement, revealing the deeper social factors that shape participation and health behaviours.

#### 4.1.5 Theme 5—Cultural Stigma, Silence, and Shifting Perspectives

Participants described how cancer remained a taboo topic in many communities, often associated with fear, secrecy, and fatalism. Several participants referred to the perception of cancer as a *death sentence* (P003, P006, P008, P009), with P008 noting that *Cancer equals death in the Black community in a lot of cases… I am defying a lot of my friends' outlooks because I am not depressed… but* that is *the norm.* Similarly, P003 explained that this stigma is rooted in limited awareness and historical underdiagnosis:

It is perceived as a death sentence because it is not something we grew up knowing about… some of them might have been cancer because there was no equipment to detect or point out exactly the cause.

Silence around cancer was also common, with P002 stating that ‘It [cancer] is not something people talk about happily… people probably discuss it in their own homes, but it isn't something that people would discuss about outside.’ Among Asian families, this secrecy could extend even within households, as P010 reflected:

For Asian families, they don’t want to tell their elderly that they have cancer… they know that once they have cancer, their family life changes.

While stigma persisted, some participants observed positive cultural shifts. P004 noted that ‘at one time they [the community] would look down on you… it's getting a lot better now because they're standing up now… it's changing,’ suggesting growing openness and resilience, particularly among Caribbean men. P005 similarly felt that ‘people are more interested in looking after themselves to avoid cancer,’ pointing to an emerging health consciousness within some communities.

Alongside stigma, participants often expressed pride in their cultural identity and heritage. P004 and P009 both spoke at length about their personal and family histories, situating their experiences of cancer within broader narratives of migration and belonging. This sense of pride and self‐definition provided valuable context for their perspectives and helped foster rapport during interviews, allowing participants to speak in their own terms about how ethnicity shaped their outlook.

Overall, this theme highlights how cultural stigma, silence, and identity intersect to shape how cancer is perceived and discussed. While stigma continues to limit open dialogue in some communities, generational shifts, increased awareness, and opportunities for representation in research appear to be slowly transforming these conversations.

The continued stigma surrounding cancer within African, Caribbean, and South Asian communities has been widely reported [47, 48, 49]. The “death sentence” narrative identified in our data echoes these findings but also demonstrates signs of cultural evolution. Caribbean participants in particular described increased willingness to speak openly about prostate cancer, suggesting that positive survivorship narratives and community champions can erode taboo and normalise discussion [50]. This theme reinforces that stigma reduction efforts must be culturally embedded, sustained, and co‐led by community members rather than delivered through top‐down campaigns.

As cultural silence around cancer begins to shift, many participants expressed a growing sense of openness and purpose, transforming stigma into motivation to help others. This desire to contribute to change and leave a positive legacy underpinned a willingness to engage in research and PA initiatives.

#### 4.1.6 Theme 6—Altruism, Legacy and Motivation to Engage

Across participants, a strong sense of altruism and purpose emerged as a key motivator for engaging in research and PA. Many viewed participation as a way to *help other* (P007) and to improve representation for those historically excluded from research. P002 reflected that *Just the fact that someone is doing it… that's enough motivation for me, the fact you have used your time to look into it, into what you're doing now for the benefit of people who have not been represented,* while P005 expressed that *Motivation to help out our community is a big factor for me.*

For several participants, involvement in research was linked to legacy and a moral duty to create change. P004 described how his diagnosis had reshaped his outlook:

One time I wasn’t thinking about doing anything like this [taking part in research] but now because I have prostate [cancer] and having this problem, so yes if I can help in any way you know.

Similarly, P008 emphasised a desire for systemic progress, noting that ‘There needs to be some sort of change… an introduction of healthcare about fitness care and how to keep a positive mindset.’

Others were driven by a belief in scientific progress and the need for diverse participation. P003 stressed that *It's always good to have a diverse group so the trial can reflect various ethnicities that exist,* highlighting a sense of responsibility to ensure their communities were visible in research. P006's engagement was also grounded in curiosity and conviction:

For that [PSA] level to be cancerous is convincing me that this is the area I would like you to research.

For some, the motivation was both communal and personal. P009 explained that *Mostly I look at the benefits for my health and how it can make me recover much more faster,* while P001 saw altruism and appreciation as intertwined, stating that *Incentives would be a nice bonus, but it's just as good to be able to help the research.* Others, such as P010, wanted to actively contribute to research delivery itself, offering:

If you have any idea, I will help you to achieve it, yeah.

Collectively, these accounts reveal that participation was rarely driven by individual benefit alone. Instead, men framed involvement as an act of giving back and transforming personal adversity into community contribution. Research and PA could become spaces not only for recovery but for empowerment and visibility, demonstrating how altruism and representation intersect in shaping engagement among men from diverse ethnic backgrounds.

Altruism has been consistently identified as a motivator for minority ethnic participation in health research [51, 52]. Our study nuances this by highlighting that altruism was often tied to representation and collective advancement rather than individual benefit. Participants' desire to “help others” for the benefit of people in their community parallels Skakoon‐Sparling et al. [53] notion of “communal altruism,” where the practice of safe‐sex in sexual minority men promotes the well‐being of their entire community. This finding suggests that recruitment strategies explicitly emphasising contribution to community wellbeing and increased representation may resonate more strongly than those centred on personal outcomes.

### 4.2 Practical Implications

As a result of the rich data, there is key learning and clear recommendations for practice.

### 4.3 Clinical Practice

The findings highlight opportunities for improving the integration and delivery of PA support across the prostate cancer care pathway. PA was described as important to men's sense of identity, recovery, and mental renewal, yet access to structured guidance was inconsistent and often culturally detached. Embedding PA into routine consultations, survivorship clinics, and follow‐up care, rather than as an optional extra, could help normalise PA as an essential component of rehabilitation.

Clinicians should receive targeted training to communicate PA guidance in ways that are both culturally sensitive and contextually relevant, aligning with recent shifts in medical education that incorporate structured cultural‐competence training [54]. This includes recognising the different meanings attached to PA across cultural groups, using examples that resonate with patients' lived experiences, and promoting informal as well as structured PA. Representation within healthcare teams was also seen as vital. Increasing workforce diversity and providing cultural competency training may enhance trust and openness, particularly among communities who have historically felt excluded from care.

Finally, developing partnerships between oncology services, community organisations, and faith or cultural centres could enable delivery of accessible, localised PA programmes. Such collaborations would not only reduce travel and cost barriers but also make PA support more visible, familiar, and sustainable for minority ethnic men living with prostate cancer.

### 4.4 Research Practice

From a research perspective, the findings underscore the importance of designing studies that are visibly inclusive, trustworthy, and relational. Participants consistently expressed willingness to engage in research when they felt the study's purpose was transparent, the team was relatable, and the benefits were clearly communicated. Recruitment should therefore prioritise face‐to‐face engagement in community and clinical settings, supported by bilingual materials and culturally representative staff.

Partnerships with trusted community figures, such as religious leaders and cultural advocates, could play a crucial role in connecting researchers with underrepresented groups. This would allow for researchers to build a sense of visibility and belonging into the recruitment process itself for potential participants. Researchers should also consider the value of peer advocates or patient ambassadors, whose lived experience can bridge the gap between academic institutions and the communities they aim to reach.

Beyond recruitment, inclusivity should extend into study design and dissemination. Co‐producing research materials, ensuring that language and imagery reflect participants' identities, and feeding findings back into communities are all essential to building trust and accountability. In this way, culturally sensitive and participatory approaches can transform research from a process of data collection into one of collaboration and empowerment that ensures diverse voices genuinely shape the future of cancer care and PA science.

### 4.5 Theoretical Implications

#### 4.5.1 Overcoming Barriers to Physical Activity Among People With Cancer From Minority Ethnic Backgrounds

Participants identified a range of intersecting barriers to PA, spanning practical, cultural, and relational domains. Common challenges included travel distance and cost, time burden and convenience, language barriers, and generational differences in attitudes toward PA. In addition, there was a sense of distrust or scepticism toward research and healthcare institutions, potentially shaped by a perceived lack of representation and culturally attuned engagement. Addressing these barriers therefore requires both structural accessibility and relational trust‐building to ensure that PA participation is equitable across all ethnic backgrounds.

To overcome logistical barriers, PA programmes should be delivered within communities rather than solely through hospital settings which is strongly encourage by the NHS Long Term Plan [55]. Locating sessions in local venues, such as community centres, mosques, churches, and leisure facilities, can reduce travel burden and cost while fostering a greater sense of belonging. Flexible scheduling, weather‐appropriate indoor options, and online or home‐based alternatives could further alleviate time and convenience pressures. Providing bilingual facilitators and culturally aware staff can help bridge communication gaps and make sessions feel welcoming and familiar.

Cultural attitudes toward PA varied across ethnic groups. Men of Caribbean heritage often viewed PA as an ingrained part of their identity. For these men, PA was associated with community, rhythm, and pride, and not perceived as burdensome. In contrast, some participants from African, Asian, and Middle Eastern backgrounds expressed less familiarity or comfort with structured PA, reflecting differing cultural norms, climate, and generational values. Recognising and learning from these positive examples, where PA is already culturally embedded, could inform the design of programmes that draw on existing community strengths and cultural practices rather than imposing external models of PA.

Building trust remains central to addressing deeper barriers. Participants consistently emphasised that face‐to‐face contact is the most trusted and effective form of engagement, while leaflets, emails, and impersonal outreach were largely dismissed. Establishing trust requires visible diversity within program staff and transparency around purpose, benefits, and outcomes. Participants were more open to engaging when they understood how PA or research participation would directly benefit themselves or their communities. Incorporating peer advocates and patient ambassadors, particularly those from the same cultural backgrounds, can help to bridge mistrust and counter scepticism in ‘the system’, while normalising participation through relatable voices.

Ultimately, overcoming barriers to PA requires a dual focus: addressing structural and linguistic challenges while cultivating representation, visibility, and trust. PA programmes and research initiatives must be co‐designed with communities, delivered in culturally meaningful spaces, and led by relatable facilitators. By drawing inspiration from the groups that already hold strong PA identities, and extending that accessibility and inclusivity to others, these efforts can transform PA into an equitable, empowering component of survivorship for all.

**FIGURE 2:**
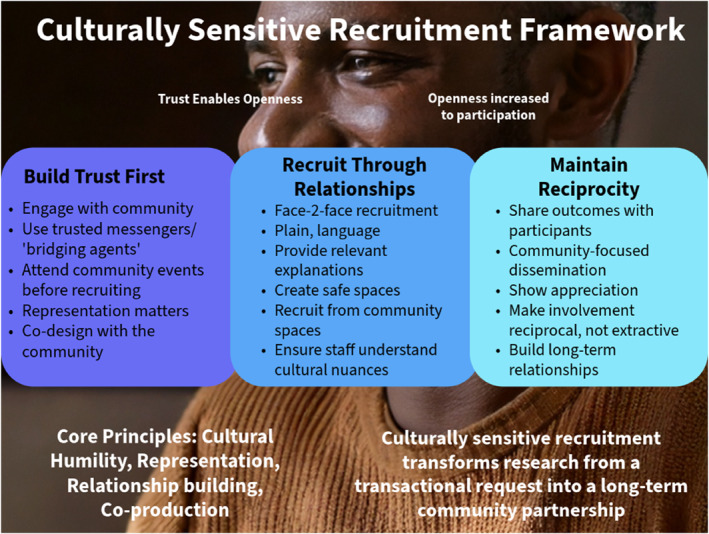
Culturally sensitive recruitment framework derived from participant experiences.

Drawing together findings across all six themes, we developed a Culturally Sensitive Recruitment Framework (See Figure 2) that synthesises insights from the six themes and participant recommendations, outlining practical principles for improving recruitment of men from minority ethnic backgrounds into exercise and clinical research.

### 4.6 Strengths and Limitations

This novel study provides insights into how men from minority ethnic backgrounds who have lived with a PCa diagnosis experience PA and research participation. By capturing first‐hand accounts across diverse cultural groups, this research extends existing understanding of PA behaviour and research engagement beyond predominantly White cohorts. The use of semi‐structured interviews using a piloted and tested topic guide allowed for deep exploration of the personal, social, and cultural meanings attached to PA, providing context‐specific insights that can inform more inclusive health and research practices. Finally, our sample size was sufficient as we reached saturation.

Several limitations must be acknowledged. Two participants did not complete the baseline questionnaire, limiting the completeness of demographic data and highlighting the use of digital data collection as a barrier to research participation. While the sample included diversity in ethnicity and life experience, all participants were men with PCa, which may restrict transferability to other urological cancers or to women. Additionally, although efforts were made to ensure reflexivity and cultural sensitivity during interviews, interpretations may still reflect the researchers' own positionalities.

## 5 Conclusion

In conclusion, this study demonstrates that PA and research participation among men with PCa from minority ethnic backgrounds are shaped by intersecting cultural, structural, and historic factors. While PA served as a vital source of continuity, mental renewal, and self‐worth, fragmented support systems and culturally insensitive care repeatedly disrupted engagement. Barriers to research participation were not due to lack of interest, but to longstanding issues of representation, accessibility, and trust.

Despite these challenges, participants voiced strong communal altruistic motivations and a desire to ensure that future generations receive better care and visibility. These findings indicate that meaningful improvements in PA support and research inclusion will require approaches that are relational, culturally grounded, and embedded within communities rather than imposed through existing institutional models. If cancer care and research are to be equitable, culturally grounded PA support and community‐led recruitment must become standard practice rather than supplementary initiatives.

### 5.1 Future Research Directions

Building on the need for culturally grounded and community‐embedded approaches identified in this study, future research should prioritise co‐produced designs that meaningfully involve community members throughout all stages of the research process. This includes broader recruitment across cancer types, greater gender diversity, and sustained partnerships with community organisations to ensure relevance, trust, and accountability.

Future work should also focus on developing and evaluating interventions that integrate PA support with culturally sensitive recruitment and communication strategies. Collaboration with faith leaders, local organisations, and patient advocates may be particularly effective in enhancing trust and engagement among underrepresented groups. Longitudinal qualitative and mixed‐methods studies are needed to explore how attitudes toward PA and research participation evolve across the cancer trajectory, from diagnosis through survivorship. Finally, embedding and systematically evaluating culturally tailored PA interventions within oncology care pathways represents a critical next step toward addressing inequities in access to supportive cancer care.

## Author Contributions

Ruth E.M Ashton conceptualised the study. Jack Carr conducted the recruitment, interviews, analysed the data gathered, and draughted the manuscript. David Broom, Mark A. Faghy, Ruth E.M Ashton and Clare Roscoe contributed to reviewing, quality checking, and refining the manuscript. Clare Roscoe contributed to draughting the topic guide with Jack Carr and Ruth E.M Ashton. Kevin Williams contributed to recruitment with Jack Carr.

## Funding

This work was supported by The Urology Foundation through a Small Project Award. The funder had no role in the study design, data collection, analysis, interpretation of findings, or the decision to submit the manuscript for publication.

## Conflicts of Interest

The authors declare no conflicts of interest.

## Supporting information


**Table S1:** Barriers to inclusivity in prostate cancer and exercise research—Topic questions.

## Data Availability

The data that supports the findings of this study are available in the supplementary material of this article.
