# Supplementary material for: Physical Activity, Trust, and Research Participation Among Men From Minority Ethnic Backgrounds Living With Prostate Cancer: A Qualitative Study
Source: Psychooncology. 2026 Feb 24;35(2):e70408. doi: 10.1002/pon.70408 (PMC12932258; doi:10.1002/pon.70408)
Supplement: Supplementary file 1 — Table S1: Barriers to inclusivity in prostate cancer and exercise research—Topic questions. [file PON-35-e70408-s001.docx]

**Supplementary Material**

**Table S1 - Barriers to inclusivity in prostate cancer and exercise research – Topic questions**

**Themes/ sections:**

1. Current exercise and physical activity levels and opinions on exercise.
2. Use and importance of exercise in cancer care pathway.
3. Perceptions and experiences of being approached to or taking part in clinical trials.
4. Barriers to taking part in clinical trials.
5. Anything that would encourage individuals to participate in clinical trials, especially those involving exercise i.e. Facilitators and Motivators.

| **Potential question** | **Theme/ section** |
| --- | --- |
| What does exercise mean to you? | 1 |
| What comes to mind when you hear the term ‘exercise’ or ‘physical activity’? | 1 |
| How physically active are you currently? | 1 |
| Do you think you meet the governments exercise recommendations? | 1 |
| (If participant is physically active) What type of exercise is enjoyable for you? | 1 |
| What about exercise do you like/ find enjoyable? | 1 |
| (If participant is not physically active) Have you ever been very physically active at a point in your life? | 1 |
| At your most physically active, how often were you exercising? | 1 |
| What exercise did you enjoy? | 1 |
| What about exercise don’t you enjoy? | 1 |
| What about exercise did you enjoy? | 1 |
| When you were diagnosed, how much information were you given on the potential risks and benefits of exercise? | 2 |
| Did your oncologist place much, if any, stress on the importance of exercise in your cancer care pathway? | 2 |
| Did your nurses place much, if any, stress on the importance of exercise in your cancer care pathway? | 2 |
| If exercise was used as a part of your cancer care, how was it delivered to you? | 2 |
| What type of exercise was used during your cancer care treatment? How long did you exercise for? How intense was the exercise? | 2 |
| (If exercise wasn’t used in treatment pathway) Do you think exercise would’ve been useful and/ or feasible during your cancer treatment pathway? | 2 |
| Do you think you might’ve benefitted more from rehabilitation or prehabilitation exercise treatment? | 2 |
| Are you aware of the potential physical and psychological benefits of exercise on cancer treatment outcomes? | 2 |
| If you were offered an exercise plan alongside your treatment plan, would you have engaged with it? | 2 |
| What would’ve been the best way to deliver exercise to you to get the best results? | 2 |
| Are there any forms of exercise you feel you would’ve been able to do during your treatment? (e.g. walking training, swimming) | 2 |
| Were there any points during your treatment where exercise would’ve been more tolerable compared to others? (e.g. 24hrs before chemo vs 24hrs after chemo infusion) | 2 |
| Were you ever approached to take part in a clinical trial during your treatment? | 3 |
| Did you take part in a clinical trial during your treatment? | 3 |
| (If you were approached) why did you not take part/ why did you take part in clinical trials? | 3 |
| If you were approached, would you have taken part? Why? Why not? | 3 |
| (If they have taken part in clinical trials) What was your experience of the clinical trial you were involved in? | 3 |
| Are you more or less likely to take part in a drug trial compared to an exercise-based trial? | 3 |
| What was positive about your clinical trial experience? | 3 |
| What could’ve been improved about your clinical trial experience | 3 |
| Are there any barriers that made taking part in exercise challenging for you? | 2 |
| Are there any barriers that made participating in clinical trials difficult? | 4 |
| Would you have wanted to take part in a clinical trial; but were never approached? | 3 |
| If so, how would’ve been the best way to approach you? | ¾ |
| Would you have preferred the recommendation for clinical trial opportunities coming from your oncologist/ nurse or from a clinical researcher? | 3 |
| Would you have preferred to have been contacted through a support group/ agency? | ¾ |
| What more could we do to encourage you to take part in clinical trials? | 5 |
| If travel costs were subsidised, would that increase the likelihood of you taking part in clinical trials? | 5 |
| Would you prefer for clinical trial sessions to be booked on the same day you have appointments at the hospital for purpose of ease? | 5 |
| Would you prefer clinical trial sessions to be held at the hospital or on university campus? | 5 |
| Is there anything that we can do to make you more comfortable when taking part in clinical trials involving exercise in particular? | 5 |
| Are you more or less likely to take part in a clinical trial that involves exercise? | 4 |
| Can you describe your current exercise/ physical activity routine/ habits/ average day in the life? | 1 |
| Did you engage in any form of physical activity during your cancer experience? | 2 |
| Did you notice any physical or emotional benefits from engaging in exercise during your cancer experience? | ½ |
| Have you/ did you struggle to maintain/ start an exercise programme since your diagnosis? | 1 |
| How do you perceive the importance of exercise regarding your cancer treatment journey? | 2 |
| Are there any support/ resources that could’ve helped incorporate exercise into your cancer care? | 2 |
| Do you have any concerns or fears related to clinical trials? | 4 |
| Are there any specific benefits or incentives that would make participation in clinical trials more appealing to you? | 5 |
| What role do support/ advocacy groups play in encouraging participants in clinical trials? | 5 |
